# Effect of Nursing Behavioral Intervention on Subjective Sleep Quality Among Postpartum Women: A Quasi-Experimental Study

**DOI:** 10.3390/healthcare14101331

**Published:** 2026-05-13

**Authors:** Eman Elsayed Hussein Mohammad, Mona Abdullah Mohamed Ali, Mahmoud Abdelwahab Khedr, Asmaa Mohamed Ali AlAbd, Salwa Ali Marzouk, Sara Farhan Alenizi, Aziza Ibrahim Mohamed, Nadia Abd ElHamed Eltohamy, Aida Ahmed Mohamed, Bataa Mahmoud Mohamed, Thuria Edrees Alhassan Fadlalla, Shimaa Salah Elsayed

**Affiliations:** 1 Department of Nursing, College of Applied Medical Sciences, University of Bisha, Bisha 67714, Saudi Arabia; ehussein@ub.edu.sa (E.E.H.M.); or aziza.hassan@fnur.edu.bu.eg (A.I.M.); thomyssara@gmail.com (T.E.A.F.); 2 Medical Surgical Nursing Department, Faculty of Nursing, Zagazig University, Zagazig 44519, Egypt; aida_zu7@yahoo.com; 3 Department of Medical-Surgical Nursing, College of Nursing, Qassim University, Buraydah 52571, Saudi Arabia; mon.ali@qu.edu.sa; 4 Psychiatric and Mental Health Nursing Department, Faculty of Nursing, University of Alexandria, Alexandria 21527, Egypt; 5 Psychiatric and Mental Health Nursing Department, Faculty of Nursing, Benha University, Benha 13511, Egypt; asmaa.elabd@fnur.bu.edu.eg (A.M.A.A.);; 6 Maternal and Child Health Department, College of Nursing, University of Hail, Hail 81481, Saudi Arabia; salwaali2014@gmail.com; 7 Pediatric Nursing Department, Faculty of Nursing, Assiut University, Assiut 71511, Egypt; 8 Psychiatric and Mental Health Nursing Department, College of Nursing, University of Hail, Hail 81481, Saudi Arabia; sara.f9a@gmail.com; 9 Faculty of Nursing, Benha University, Benha 13511, Egypt; 10 Maternal and Newborn Health Nursing, Faculty of Nursing, Helwan University, Cairo 11795, Egypt; nadia_mohammed@nursing.helwan.edu.eg; 11 Nursing Program, Fakeeh Care Group, Nursing Department, Fakeeh College for Medical Sciences, Jeddah 21461, Saudi Arabia; 12 Nursing Department, North Private College of Nursing, Arar 73312, Saudi Arabia; coating_1@yahoo.com; 13 Pediatric Nursing Department, Faculty of Nursing, Zagazig University, Zagazig 44519, Egypt

**Keywords:** behavioral education, sleep quality, postpartum women, quasi-experimental

## Abstract

Background: Sleep disturbances are linked to adverse outcomes in postpartum women. While behavioral interventions can potentially improve sleep, research in this area during the postpartum period is limited. This study aims to evaluate the effect of a behavioral education program on sleep quality among postpartum women. Methods: A quasi-experimental design with a pretest–posttest approach was employed, including a control group for comparative analysis. The research was conducted at the Maternal and Child Health Center in Egypt, focusing on the sleep disturbances experienced by postpartum women. The study involved 280 postpartum women (140 in the study group and 140 in the control group) who had given birth within the last two months. Results: Post-intervention, the study group demonstrated significant improvements in all components of sleep quality, with *p*-values < 0.001. Conversely, the control group experienced a deterioration in sleep quality, with 77.9% reporting poor sleep quality post-intervention. Conclusion: The behavioral education program significantly enhanced sleep quality among postpartum women, suggesting that such interventions should be integrated into maternal healthcare practices to improve postpartum sleep management.

## 1. Introduction

Sleep disturbances are a common global concern among postpartum women and are associated with adverse physical, psychological, and emotional outcomes. The postpartum period or puerperium starts after placental delivery and finishes when the mother’s physiological and anatomical systems return to their non-pregnant condition [1,2]. This period is typically classified into three stages: acute (0–24 h after delivery), early (up to 1 week), and late (up to 6 weeks or up to 6 months), with each stage associated with different clinical and psychosocial difficulties [3].

Pregnancy and the postpartum period are times of significant physical, psychological and social changes; sleep disturbances are common during this time [4,5]. New mothers commonly receive inadequate and interrupted sleep, especially during the first 6 weeks postpartum, when hours of total sleep per night average about six hours [6]. Sleep disturbances can present as a sleep deficit with shortened total duration or fragmented sleep with multiple nocturnal awakenings, leading to poor continuity of sleep [7]. Chronic sleep disturbance has been linked with irritability, lack of focus, lower general health-related quality of life, postpartum depression, anxiety, and other negative effects on both physical and mental health [8,9]. Adequate sleep is essential for physical recovery, hormonal regulation, and emotional stability during the postpartum period [8].

There is a high prevalence of sleep problems in the postpartum period, yet effective treatment options are scarce in clinical practice. While sleep disturbances can be perceived as a normal part of caring for an infant, clinicians are not commonly equipped with evidence-based interventions for promoting maternal sleep health during the early postpartum stage [10]. As sleep of the mother and infant is highly important, treating maternal sleep disturbances is important for both maternal health as well as mother–infant interaction.

Behavioral and educational interventions are one of the pillars of preventive healthcare and have proven effective in sleep outcomes across populations [11,12]. Nonpharmacological methods, particularly cognitive–behavior therapy for insomnia (CBT-I) and sleep hygiene education (SHE), are designed to alter maladaptive sleep-related behaviors and beliefs via techniques such as stimulus control, sleep restriction, relaxation strategies, and restructuring of cognitions [13,14,15]. Although CBT-oriented interventions work well, treatment should be specific to the woman’s condition, clinical needs, and medical history, and should be administered by health professionals trained in the procedure [16,17]. Nurses are in a unique position to deliver structured educational and behavioral interventions due to their continuous contact with postpartum women [2]. Integrating such interventions into routine maternal and child health services may enhance accessibility and effectiveness [1].

Despite the growing evidence on sleep interventions, there is limited research focusing on early postpartum women, particularly within routine maternal healthcare settings. The majority of studies concentrate on pregnancy or late postpartum periods and there is a dearth of literature about early postpartum periods when sleep disruption is usually at its peak, and supportive services are limited. In addition, relatively little research has been conducted to date into structured behavioral sleep interventions implemented in mainstream maternal and child health settings during this critical period. Consequently, the purpose of this study is to fill that gap by examining the impact of a structured behavioral education intervention on sleep disturbances in postpartum women during the late postpartum period.

### 1.1. Significance of the Study

New mothers are more likely than non-postpartum women to experience sleep disturbances, with exhaustion peaking during the initial three months after childbirth. Sleep disruption during the first portion of the postpartum period is prevalent (70.5% to 72.3%) [18]. A study conducted in Cairo’s Ain Shams Maternity Hospital’s postpartum ward in Egypt found that 45.2% of Egyptian mothers had sleep difficulties [19].

The most frequent causes of sleep disorders are linked to the feeding and sleeping habits of newborns. Postpartum women’s poor sleep and altered circadian rhythm are caused by a quick decline in placental hormones after birth and the newborn’s irregular sleep patterns [20]. Addressing this gap is essential to improving maternal health outcomes and supporting evidence-based nursing practice. In this context, educating postpartum women about different strategies to manage these disturbances and promote healthy sleep habits is crucial. Therefore, this study aimed to evaluate the effect of a nursing behavioral program on sleep quality among postpartum women.

### 1.2. Research Hypotheses

The following research hypotheses were developed to achieve the study’s aim:

**H0.**  *There is no difference between the control and study groups’ sleep quality before and after the behavioral education program.*

**H1.**  *Compared to the control group, the study postpartum women will have better sleep quality after completing a behavioral education program.*

## 2. Methods

### 2.1. Research Design

This study employed a quasi-experimental pretest–posttest non-equivalent control group design to evaluate the effect of an educational intervention on postpartum sleep disturbance. The design included two groups: an intervention group that received the educational program and a control group that received routine postnatal care. Both groups were assessed at two time points, namely baseline (pre-intervention) and post-intervention. Participants were allocated to the groups without randomization; therefore, the design does not meet the criteria of a randomized controlled trial. Participants were assigned to the intervention and control groups based on recruitment order and scheduling considerations to facilitate the implementation of the educational sessions and avoid contamination between groups. Baseline comparability between the two groups was assessed to minimize potential selection bias.

### 2.2. Study Setting and Participants

The study was conducted at the Maternal and Child Health (MCH) Center in Benha City, Egypt, which is affiliated with the Ministry of Health. The center provides a range of maternal and child healthcare services, including vaccination clinics, postpartum follow-up care, health education services, and outpatient consultations. Approximately 700–800 postpartum women attend the center monthly for infant immunization and routine postnatal follow-up services, with an estimated total of about 4500 women attending the center during the study period.

Participants were recruited using a convenience sampling method from postpartum women attending the vaccination clinic during the data collection period. Women were approached individually and screened to ensure that they met the inclusion criteria and had no exclusion conditions.

Women were eligible for inclusion if they were postpartum women aged 18 years or older, were less than six weeks postpartum at the time of recruitment, had a healthy breastfeeding infant, were medically stable, and agreed to participate by providing written informed consent. In this study, medically stable referred to the absence of acute maternal complications requiring hospitalization or urgent medical intervention. A healthy infant referred to a full-term infant without congenital anomalies, chronic illnesses, or extended hospitalization.

Women were excluded if they had physician-diagnosed medical or psychiatric conditions that could affect sleep patterns. These conditions included chronic pain disorders, thyroid dysfunction, anemia, diabetes mellitus, major depressive disorder, anxiety disorders, or other health conditions known to influence sleep quality. Women were also excluded if they or their infants had complications requiring prolonged hospitalization or if they were using medications known to affect sleep patterns, such as sedatives, antidepressants, or corticosteroids.

The participants were informed that participation was voluntary and that they could withdraw from the study at any time without any penalty. Women who withdrew from the study or did not complete the post-intervention assessment were excluded from the final analysis.

### 2.3. Sample Size

The sample size was calculated using the standard formula for estimating a population proportion with finite population correction. The calculation assumed a 95% confidence level and a 5% margin of error. The estimated prevalence of poor sleep quality among postpartum women was based on previous literature and was set at 24% based on previous literature [21].

Based on these assumptions and a total population of approximately 4500 postpartum women attending the Maternal and Child Health Center during the study period, the required sample size was estimated to be 280 participants. The participants were equally distributed into two groups, with 140 women assigned to the intervention group and 140 to the control group.

### 2.4. Study Variables

The study included several variables related to postpartum women. Sociodemographic and obstetric characteristics such as maternal age, educational level, marital status, employment status, family income, parity, and number of living children were collected as independent variables. Maternal sleep quality during the postpartum period was considered the primary outcome variable.

### 2.5. Instruments

Data was collected using a structured interviewing questionnaire developed by the researcher based on an extensive review of relevant literature. The questionnaire consisted of two main sections. The first section assessed the sociodemographic and obstetric characteristics of the participants, including maternal age, educational level, marital status, employment status, family income, parity, and number of living children.

The second section assessed maternal sleep quality using the Pittsburgh Sleep Quality Index (PSQI) [22]. The PSQI is a standardized self-rated instrument consisting of 19 items that evaluate sleep quality over the previous month. The instrument measures seven components: subjective sleep quality, sleep latency, sleep duration, habitual sleep efficiency, sleep disturbances, use of sleep medication, and daytime dysfunction. Each component is scored on a scale ranging from 0 to 3, and the component scores are summed to produce a global score ranging from 0 to 21. The PSQI component scores are categorical and do not directly represent actual values such as sleep duration in hours or sleep latency in minutes. Higher scores indicate poorer sleep quality. A global PSQI score of 5 or greater indicates poor sleep quality [23]. For the purposes of this study, the PSQI global score was used as the quantitative measure of postpartum sleep disturbance. The questionnaire was reviewed by a panel of experts in maternal and postpartum nursing to ensure content validity. The Pittsburgh Sleep Quality Index (PSQI) was translated into Arabic using forward and backward translation procedures. The translated version was reviewed by experts in nursing and medicine to ensure clarity, accuracy, and cultural appropriateness. The Arabic version of the PSQI has demonstrated acceptable reliability in previous studies, with Cronbach’s alpha values ranging between 0.70 and 0.85 [22,24,25,26].

### 2.6. Data Collection

Data were collected over a period of ten months from mid-July 2023 to mid-May 2024 at the vaccination clinic of the Maternal and Child Health Center. Eligible postpartum women attending the clinic were approached individually and informed about the purpose of the study. Women who agreed to participate provided written informed consent before participation. Data were collected through face-to-face interviews using a structured questionnaire administered in Arabic. The interviews were conducted by trained nursing researchers who were available to clarify any questions raised by the participants. Each interview lasted approximately 15–30 min and was conducted in the waiting area of the clinic while ensuring the participants’ privacy and confidentiality. Baseline data included sociodemographic characteristics and sleep quality assessment using the Pittsburgh Sleep Quality Index. The same instrument was used again after the intervention to evaluate changes in sleep quality.

### 2.7. Study Procedures

The study procedures were conducted sequentially at the Maternal and Child Health Center and consisted of a baseline assessment, implementation of the educational intervention, and a post-intervention evaluation. At baseline, eligible postpartum women from both the intervention and control groups completed the structured questionnaire and the Pittsburgh Sleep Quality Index. A baseline assessment was performed prior to the educational intervention. The participants in the intervention group attended four structured educational sessions conducted in small groups of five to six women at the Maternal and Child Health Center. Sessions were scheduled twice weekly and each session lasted approximately 45–60 min. The baseline assessment was conducted within the first six weeks postpartum prior to the intervention. The educational intervention was delivered over a two-week period. The post-intervention assessment was conducted at approximately two months postpartum during the routine infant immunization visit.

All sessions were delivered by the same trained nursing researchers who received prior standardized training. A structured intervention protocol was followed to ensure consistency and maintain intervention fidelity across all sessions. All sessions followed the same standardized structure and teaching strategies to ensure the consistency of the intervention. Teaching methods included short lectures, visual presentations, video demonstrations, guided group discussions, and distribution of illustrated Arabic educational booklets. Each session began with a brief review of previous content and concluded with interactive discussion and reinforcement of key educational messages.

The first session introduced participants to the concept of postpartum sleep and normal changes in sleep patterns following childbirth, as well as common postpartum sleep disturbances and their effects on maternal well-being. The second session focused on factors affecting sleep quality during the postpartum period, including hormonal changes, infant feeding patterns, environmental conditions, and psychological stressors. The third session addressed evidence-based sleep hygiene practices such as establishing regular sleep routines, creating a comfortable sleep environment, practicing relaxation techniques, and adopting healthy lifestyle behaviors. The fourth session focused on practical coping strategies and maternal self-care practices to help women manage sleep disturbances and maintain overall well-being during the postpartum period. These strategies included relaxation techniques such as deep breathing exercises, establishing flexible sleep routines, prioritizing rest, managing night awakenings, and seeking family or social support for infant care. The participants in the control group received routine care provided at the Maternal and Child Health Center and were given a copy of the educational booklet without participating in the structured educational sessions [11,27,28].

Following completion of the educational program, post-intervention data were collected from both groups during the routine infant immunization visit at two months postpartum using the same questionnaire and the Pittsburgh Sleep Quality Index to assess changes in sleep quality. This timing reflects the routine clinical follow-up practice at the Maternal and Child Health Center.

### 2.8. Ethical Considerations

This study was approved following clearance from Benha University’s Ethical Committee (approval number: REC-PSY-N-P3). Before answering the questionnaire, all women were given a detailed explanation and asked for their voluntary agreement. Furthermore, participants were informed that there was no risk associated with participating in the research. Every participant in the study remained anonymous. Also, they were informed that the data collected would be kept confidential and utilized exclusively for research. The participants were allowed to withdraw from the study at any time.

### 2.9. Statistical Design

Data were analyzed using IBM SPSS Statistics version 21.0 (IBM Corp., Armonk, NY, USA). Descriptive statistics were calculated for all variables. Continuous variables were summarized using means and standard deviations, while categorical variables are presented as frequencies and percentages. The normality of the data was assessed using the Shapiro–Wilk test, and the results indicated that the data were approximately normally distributed. Baseline comparability between the intervention and control groups was evaluated using independent *t*-tests for continuous variables and chi-square tests for categorical variables. Between-group differences in post-intervention outcomes were assessed using independent *t*-tests. Effect sizes were calculated using Cohen’s d to determine the magnitude of the intervention effect, with values of 0.2, 0.5, and 0.8 interpreted as small, medium, and large effects, respectively. All statistical tests were two-tailed, and statistical significance was set at *p* < 0.05.

## 3. Results

### 3.1. Recruitment and Flow of Participants

**Figure 1:**
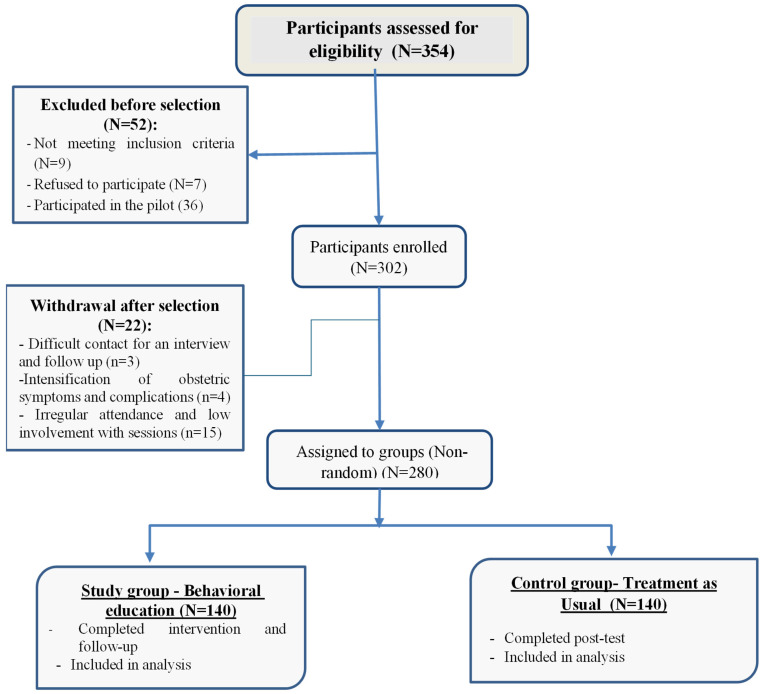
Flow diagram of participant selection for the quasi-experimental study.

A total of 354 women were assessed for eligibility. After applying the inclusion and exclusion criteria, 302 participants were enrolled in the study. After removing those lost to follow-up, the final sample consisted of 280 postpartum women. The process for selecting participants is presented in Figure 1.

### 3.2. Sociodemographic Characteristics of the Participants

A total of 280 postpartum women completed both the pre- and post-intervention assessments. The distribution of sociodemographic characteristics of participants in the control and study groups is presented in Table 1.

Regarding age, 38.6% of participants in the control group and 44.3% in the study group were between 26 and 31 years old, with mean ages of 27.98 ± 3.88 and 28.45 ± 3.56 years, respectively. With respect to educational level, half of the participants in the control group (50.0%) and 45.0% in the study group had completed secondary education.

Most participants in both groups were married (86.4% in the control group and 85.7% in the study group). Concerning occupation, 43.6% of the women in the control group and 35.7% in the study group were employed. Regarding parity, 59.3% of the women in the control group and 54.3% in the study group had experienced fewer than five deliveries. Similar distributions were observed in the number of children and family income levels.

No statistically significant differences were observed between the control and study groups across all sociodemographic variables, including age, educational level, marital status, occupation, parity, number of children, and family income, indicating that the two groups were comparable.

### 3.3. Comparison of Sleep Quality Components Between Groups Before and After the Educational Intervention

Table 2 presents the comparison of sleep quality components between the control and study groups before and after the intervention.

At baseline (pre-intervention), no statistically significant differences were observed between the control and study groups in any component of sleep quality, including subjective sleep quality, sleep latency, sleep duration, habitual sleep efficiency, sleep disturbances, use of sleeping medication, daytime dysfunction, or the total sleep quality score.

After the intervention, statistically significant differences were observed between the two groups across all sleep quality components. The study group demonstrated significantly lower mean scores, indicating improved sleep quality compared with the control group. Improvements were observed in subjective sleep quality, sleep latency, sleep duration, habitual sleep efficiency, sleep disturbances, use of sleeping medication, and daytime dysfunction (all *p*-values = 0.001).

Similarly, the total sleep quality score showed a statistically significant difference between the control group (13.89 ± 2.75) and the study group (10.04 ± 2.14) following the intervention (*p* = 0.001). Effect size analysis indicated moderate to large intervention effects across sleep quality domains (Cohen’s d = 0.50–1.56), suggesting a substantial improvement in sleep quality among women in the intervention group.

### 3.4. Distribution of Overall Sleep Quality Scores

The distribution of overall sleep quality scores before and after the intervention is presented in Table 3. Before the intervention, poor sleep quality was reported by 62.1% of participants in the control group and 64.3% in the study group, with no statistically significant difference between the groups (χ^2^ = 0.138, *p* = 0.402).

Following the intervention, the proportion of participants reporting poor sleep quality increased in the control group (77.9%) but markedly decreased in the study group (26.4%). Conversely, good sleep quality was reported by 73.6% of participants in the study group compared with 22.1% in the control group. The difference between the groups after the intervention was statistically significant (χ^2^ = 74.19, *p* = 0.001). These findings suggest that the intervention not only improved overall sleep quality but also had a positive impact on specific sleep parameters, including reduced time to fall asleep, increased sleep duration, and improved sleep efficiency.

## 4. Discussion

Although postpartum maternal sleep disruption is common, there is still a lack of research on early postpartum sleep among mothers [29]. This phase is typically defined by a considerable decrease in structured support for women from nurses and also health education, which may serve to worsen sleep issues. The present intervention directly targets this gap in current knowledge by examining the effects of behavioral education on sleep disturbance following childbirth as soon as 2 months after parturition.

Owais et al. (2018) [30] found that nonpharmacological sleep interventions improved subjective sleep quality up to eight weeks and but not beyond nine weeks postpartum. As a result, a central tenet to the design of this study was to provide postpartum women with concrete behavioral strategies for addressing sleep disturbances and improving sleep behaviors during this sensitive early phase. The intervention demonstrated an overall beneficial effect on sleep quality, as significant improvements were observed across all components of PSQI, including subjective sleep quality, sleep latency, duration of sleep, habitual sleep efficiency, sleep disturbances, use of sleeping medications, and daytime dysfunction (mean ± SD; all *p* = 0.001). These findings are consistent with previous study demonstrating the effectiveness of behavioral sleep interventions among postpartum women [31].

The range of improvements may indicate that the intervention successfully targeted several aspects of sleep hygiene and behavior, which is significant given that postpartum sleep disturbances are multifactorial in nature [9]. In contrast, Stremler et al. (2013) [10] found no significant difference in sleep endpoints between intervention and usual care arms among primiparous women. This inconsistent relationship can perhaps be explained by context and individual factors, including a more demanding caregiving environment and a higher frequency of night wakings for first-time mothers that could counteract the benefits of behavioral interventions alone.

However, previous studies suggest that postpartum sleep may be influenced by contextual and socioeconomic factors. Postpartum sleep patterns are not determined solely by biological and caregiving dynamics, but may also be shaped by broader structural and social conditions. Low income, limited education, unstable employment, and poor housing have been associated with increased psychosocial stress, reduced access to healthcare, and disrupted sleep environments. Prior research has shown that low socioeconomic status (SES) is associated with poorer sleep quality during pregnancy and across the lifespan, as well as with increased susceptibility to stress, depression, and sleep disorders [32]. However, these factors were not examined in the present analysis; therefore, their impact on the observed outcomes cannot be determined.

In the present study, there was a significant reduction in sleep latency among women belonging to the intervention group, matching the result obtained by Schroeck et al. (2016) [33] who indicated that educational programs are especially effective for decreasing sleep onset latency. These findings are further supported by those of a study at Bielefeld University in which women in late pregnancy and the first three months postpartum frequently reported longer sleep onset, frequent nocturnal wakings, as well as an increased degree of sleep problems [34]. Socioeconomic stressors, including job insecurity and household obligations, might also adversely affect sleep onset by heightening bedtime cognitive and emotional arousal. Although the intervention was associated with improved sleep quality, postpartum sleep is influenced by multiple factors, including hormonal changes, infant feeding patterns, night awakenings, caregiving responsibilities, and maternal psychological stress. Therefore, the improvement observed should be interpreted in light of these contextual factors as well as the educational intervention.

In addition to improvements in sleep latency, a significant decrease in the use of sleeping medications was observed, which indicates the positive effect of behavioral intervention on reducing reliance on pharmacological treatments among the intervention group. This finding is consistent with studies demonstrating that cognitive–behavioral therapy (CBT-I) for insomnia decreases hypnotic medication in adult population [33]. This result is particularly relevant for postpartum and breastfeeding women, for whom use of medications may pose potential health risks [35]. Furthermore, socioeconomic limitations may affect access to healthcare services and reduce opportunities for non-pharmacological support, which may further influence sleep outcomes among postpartum women.

Similarly, improvements in daytime functioning were observed; enhancements in daytime dysfunction also suggest that improved sleep resulted in better day-to-day functioning. Postpartum sleep disturbance has not only been linked to impaired maternal–infant interaction, but also increased fatigue and poorer maternal mood. Daytime sufficiency, fatigue, poor sleep quality and low resilience have been reported to be strong predictors of postpartum depression in the literature [21,36,37]. Psychosocial adversity may serve to exacerbate these effects by impeding opportunities for recovery, social support, and access to mental health services.

Furthermore, notable improvements were observed in sleep efficiency and sleep disturbance scores, indicating that the participants spent a greater proportion of time asleep while in bed during the intervention period. These findings are consistent with those of Tobback et al. (2017) [38], who reported positive subjective sleep quality among breastfeeding mothers despite reduced sleep efficiency in early postpartum. Although Sweeney et al. (2020) suggested that small improvements in sleep efficiency may have minimal clinical relevance, the observed gains in this study may still be meaningful when situated within the broader social and socioeconomic landscape of postpartum women’s lives [31].

The findings of this study have significant implications for nursing practice, particularly in maternal and post-partum care settings. Nurses are well positioned to play a key role in improving sleep quality among post-partum women through the implementation of structured educational interventions [39]. Integrating sleep hygiene education into routine postpartum care has the potential to enhance maternal well-being and reduce the risk of post-partum complications [40]. In addition, equipping nurses with training in evidence-based sleep interventions can strengthen their role in promoting maternal health outcomes. Furthermore, such interventions can be systematically incorporated into maternal and child health centers as part of standard postpartum care services [41]. The observed improvements in sleep quality may be attributed to several components of the intervention. The structured educational sessions, combined with repeated reinforcement, interactive discussions, and the use of visual and written materials, likely enhanced the participants’ understanding of sleep hygiene and encouraged behavioral changes related to it. The frequency and continuity of the sessions may have also supported adherence to the recommended practices.

In addition, the nursing-based nature of the intervention may have played a significant role in its effectiveness. Nurses are uniquely positioned to provide patient-centered care, continuous support, and individualized guidance, which may enhance participant engagement and facilitate the adoption of healthy sleep behaviors [39]. This approach distinguishes the intervention from other purely educational or self-directed strategies, as it integrates education with ongoing support and follow-up.

### Strengths and Limitations

This current study, conducted at the Maternal and Child Health Center in Benha City, Egypt, offers early evidence that a behavioral educational intervention can reduce sleep disturbances among postpartum women and provides a basis for future larger studies. The quasi-experimental design increased the internal and external validity compared to other non-experimental designs. The structure and order of treatment sessions corresponded to the participants’ learning and were delivered in a consecutive fashion with adequate time given between interventions.

Several limitations should be acknowledged. First, self-reported data was used, which may have introduced recall bias, as well as potential over- or underreporting. Second, the study protocol was occasionally prolonged due to missed appointments by some participants, which required rescheduling and may have affected consistency in the data collection.

Although it would have been preferable to implement the intervention during late pregnancy, the study focused on the postpartum period as breastfeeding support is particularly critical during this time for promoting maternal adjustment and reducing the risk of postnatal difficulties. In addition, the use of convenience sampling from a single maternal and child health (MCH) center may limit the generalizability of the findings and increase the risk of selection bias. Furthermore, participants were not randomly assigned to the intervention and control groups, which represents a potential source of selection bias and may lead to baseline differences between groups. This limitation may affect the internal validity of the study and should be considered when interpreting the causal effect of the intervention. Although sociodemographic variables were collected and showed no significant differences between the groups, the study did not include adjusted analyses to control for their potential confounding effects.

Therefore, the results should be interpreted with caution. Future studies using probability sampling, multicenter designs, and longer follow-up periods are recommended to enhance external validity. Sleep quality may be affected by several factors that were not controlled for in this study, including hormonal changes, infant feeding patterns, and caregiving demands. As the PSQI assesses sleep over the previous month, the results may reflect both pre- and post-intervention sleep patterns.

## 5. Conclusions

In conclusion, this quasi-experimental study provides compelling evidence for the effectiveness of a behavioral education program in improving sleep quality among postpartum women. The intervention demonstrated a consistent improvement across all sleep components including subjective sleep quality, sleep latency, sleep duration, habitual sleep efficiency, sleep disturbances, daytime dysfunction, and, notably, a reduction in the use of sleeping medications, supporting its effectiveness. These comprehensive improvements, reflected by the substantial decrease in the total sleep quality score for the intervention group, underscore the potential of non-pharmacological approaches in addressing postpartum sleep issues.

### Recommendations

Based on the findings of this study, it is recommended that nursing practice in maternal and postpartum care settings actively incorporate the assessment and management of sleep disturbances as part of routine care. Nurses should be encouraged to identify modifiable factors affecting sleep among postpartum women and to develop individualized care plans that support maternal recovery and overall well-being. Furthermore, there is a need for continued research to refine and evaluate evidence-based, nursing-led interventions targeting postpartum sleep disturbances. Future studies should also explore potential mediators influencing the effectiveness of these interventions, as well as assess their long-term impact on sleep quality and daytime functioning through extended follow-up. In addition, investigating the role of digital health-enabled interventions may provide innovative approaches to preventing and managing sleep problems during the postpartum period.

## Figures and Tables

**Table 1:** Distribution of personal characteristics of the study participants (N = 280).

Variable	Item	Control Group		Study Group		Chi Square	*p*-Value
		No	%	No	%		
Age in years	20–25	40	28.6	33	23.6	1.23	0.540
	26–31	54	38.6	62	44.3		
	≥32	46	32.9	45	32.1		
	Mean ± SD	27.98 ± 3.88		28.45 ± 3.56			
Educational qualification	Primary	16	11.4	26	18.6	2.83	0.242
	Secondary	70	50.0	63	45.0		
	University	54	38.6	51	36.4		
Marital status	Married	121	86.4	120	85.7	1.31	0.519
	Divorced	11	7.9	15	10.7		
	Widow	8	5.7	5	3.6		
Occupation	Yes	61	43.6	50	35.7	1.81	0.111
	No	79	56.4	90	64.3		
Parity	Primiparas	33	23.6	44	31.4	2.24	0.326
	<5 times	83	59.3	76	54.3		
	≥5 times	24	17.1	20	14.3		
Number of children	One child	33	23.6	42	30.0	1.47	0.478
	2–3 children	83	59.3	76	54.3		
	≥3 children	24	17.1	22	15.7		
Family income	Not enough	74	52.9	81	57.9	0.708	0.235
	Enough	66	47.1	59	42.1		

*p* > 0.05 not significant.

**Table 2:** Distribution of sleep quality among studied postpartum women pre- and post-intervention (N = 280).

Item	Pre-Intervention				Post-Intervention				Cohen’s d	Effect
	Control Group	Study Group	*t*-Test	*p* Value	Control Group	Study Group	*t*-Test	*p* Value		
Subjective sleep quality	1.77 ± 0.9219	1.67 ± 0.96	0.888	0.373	1.95 ± 0.84	1.10 ± 0.77	8.85	0.001 **	1.06	Large
Sleep latency	2.15 ± 0.8726	2.29 ± 0.84	1.39	0.165	2.37 ± 0.59	1.92 ± 0.93	4.87	0.001 **	0.58	Moderate
Sleep duration	2.22 ± 0.6240	2.17 ± 0.56	0.602	0.548	2.27 ± 0.64	1.97 ± 0.44	4.52	0.001 **	0.54	Moderate
Habitual sleep efficiency	1.57 ± 0.6897	1.62 ± 0.84	0.465	0.642	1.81 ± 0.61	1.44 ± 0.74	4.54	0.001 **	0.55	Moderate
Sleep disturbances	1.53 ± 0.7530	1.4429 ± 0.73	1.04	0.296	1.70 ± 0.71	1.36 ± 0.62	4.17	0.001 **	0.50	Moderate
Use of sleeping medication	1.89 ± 0.8789	1.7857 ± 0.88	1.01	0.311	2.07 ± 0.7	1.31 ± 0.64	8.94	0.001 **	1.14	Large
Daytime dysfunction	1.22 ± 0.8077	1.4286 ± 1.03	1.79	0.073	1.69 ± 0.64	0.92 ± 0.66	9.77	0.001 **	1.17	Large
Total sleep quality score	12.38 ± 2.675	12.42 ± 2.89	0.129	0.898	13.88 ± 2.75	10.04 ± 2.14	13.03	0.001 **	1.56	Large

** Highly significant (*p* value = 0.001). t: independent *t*-test.

**Table 3:** Distribution of total sleep quality score among control and study group pre- and post-intervention.

Intervention	Sleep Quality Score	Control Group		Study Group		Chi Square	*p*-Value
		No	%	No	%		
Pre-intervention	Poor sleep quality	87	62.1%	90	64.3%	0.14	0.402
	Good sleep quality	53	37.9%	50	35.7%		
Post-intervention	Poor sleep quality	109	77.9%	37	26.4%	74.19	0.001 **
	Good sleep quality	31	22.1%	103	73.6%		

** Highly Significant level at *p* value = 0.001.

## Data Availability

The data presented in this study are available on request from the corresponding author due to privacy and confidentiality concerns, and ethical guidelines restricting data distribution.
